# The *var3* genes of *Plasmodium falciparum* 3D7 strain are differentially expressed in infected erythrocytes

**DOI:** 10.1051/parasite/2014019

**Published:** 2014-04-24

**Authors:** Yana Zhang, Ning Jiang, Zhiguang Chang, Henan Wang, Huijun Lu, Mats Wahlgren, Qijun Chen

**Affiliations:** 1 Key Laboratory of Zoonosis, Ministry of Education, Institute of Zoonosis, Jilin University Changchun 130062 PR China; 2 Department of Microbiology, Tumour- and Cellular Biology, Karolinska Institutet S-171 71 Stockholm Sweden

**Keywords:** *Plasmodium falciparum*, *var* gene, PfEMP1, Expression

## Abstract

*Plasmodium falciparum* erythrocyte membrane protein 1 (PfEMP1) is an important virulence factor encoded by a family of 59 *var* genes, including 56 *var* genes plus 3 small *var3* genes. The *var* genes are among the most diverse sequences in the *P. falciparum* genome, but the *var3* genes are found conserved in most *P. falciparum* strains. Previous studies have been mainly focused on the typical *var* genes, while the biological characteristics of the *var3* genes remain unknown. In this study, the three *var3* genes, PF3D7_0100300, PF3D7_0600400, and PF3D7_0937600, were found to be transcribed in the erythrocytic stages of *P. falciparum*, with a peak in the transcription level at 16 h post-invasion, but terminated immediately after 16 h post-invasion. The encoded protein of PF3D7_0600400 could be detected in both the late trophozoite stage and schizont stage, while the encoded proteins of PF3D7_0100300 and PF3D7_0937600 could only be detected in the late trophozoite stage and schizont stage, respectively. Thus, the *var3* genes of the *P. falciparum* 3D7 strain were differentially expressed during the erythrocytic development of the parasite.

## Abstract (fr)

La protéine 1 de membrane de l’érythrocyte de *Plasmodium falciparum* (PfEMP1) est un important facteur de virulence, codé par une famille de 59 gènes *var*, qui comprend cinquante-six gènes *var* et trois petits gènes *var3*. Les gènes *var* sont parmi les séquences les plus diverses dans le génome de *P. falciparum*, mais sont sont conservés dans la plupart des souches de *P. falciparum*. Des études antérieures ont été principalement axées sur les gènes *var* typiques, tandis que les caractéristiques biologiques des gènes *var3* restent inconnues. Dans cette étude, il a été trouvé que les trois gènes *var3*, PF3D7_0100300, PF3D7_0600400 et PF3D7_0937600, sont transcrit dans les stades érythrocytaires de *P. falciparum*, avec un pic du niveau de la transcription à 16h post- invasion, qui s’arrête immédiatement après 16h post- invasion. La protéine codée par PF3D7_0600400 a pu être détectée à la fois dans le stade trophozoïte tardif et le stade schizonte, tandis que les protéines codées par PF3D7_0100300 et PF3D7_0937600 ne pouvait être détectées, respectivement, qu’au stade trophozoïte final et au stade schizonte. Les gènes *var3* de la souche 3D7 de *P. falciparum* sont donc exprimés de façon différentielle au cours du développement érythrocytaire du parasite.

## Introduction

*Plasmodium falciparum* malaria is still a major threat to human life and health, especially in sub-Saharan Africa [1]. An estimated 300–500 million clinical cases occur each year, with 660,000 deaths [1]. It is a disease that mainly affects immunologically naive individuals, especially children under 5 years old, and women who are in their first or second pregnancy [5, 9]. Malaria vaccine development has been hampered by the frequent antigenic variation of the *P*. *falciparum* parasites. *Plasmodium falciparum* erythrocyte membrane protein 1 (PfEMP1) is one of the most extensively studied variant surface antigens. Members of the PfEMP1 family mediate the cyto-adherence of infected erythrocytes to host receptors, allowing the parasites to avoid splenic clearance, and the tremendous sequence variation within the variant family has enabled the parasites to escape host immune responses [2, 17].

**Figure 1.**
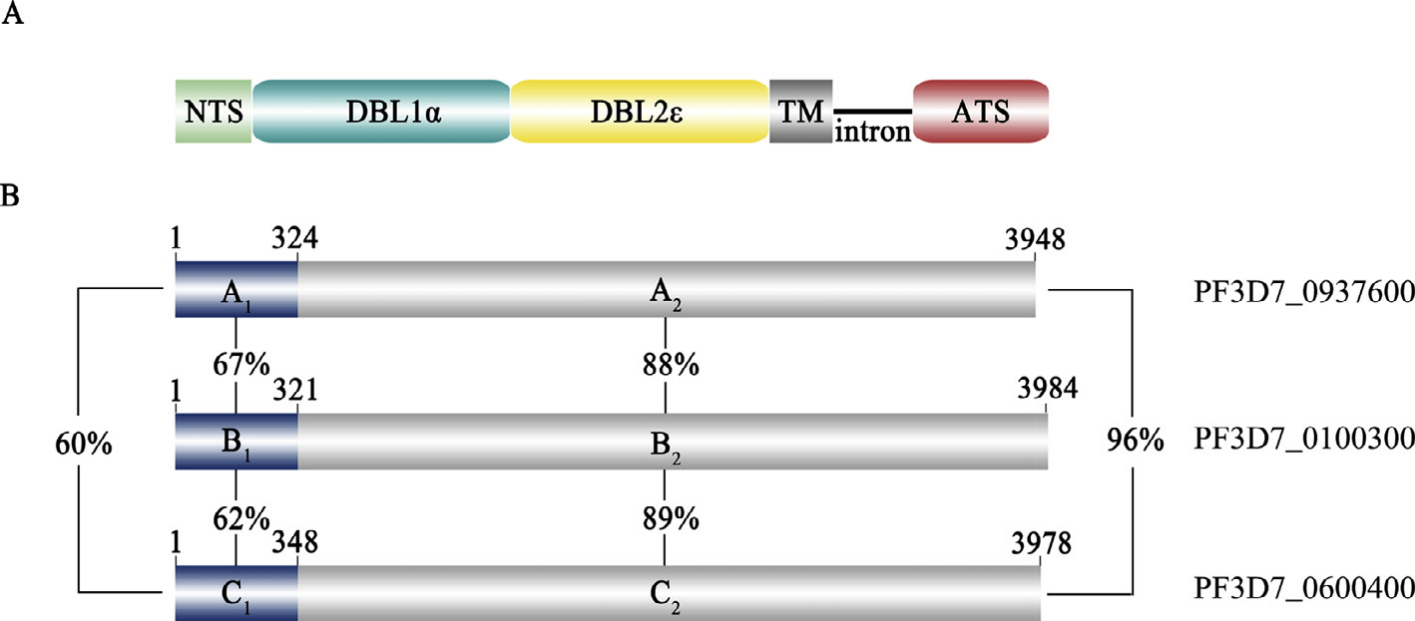
Schematic structures of the three *var3* genes and the encoded proteins. A. The domain architecture of the “min-PfEMP1s” encoded by the three *var3* genes. NTS: *N*-Terminal Segment; DBL: Duffy Binding-Like; ATS: Acidic Terminal Segment; TM: Transmembrane region. B. The variable (A1, B1, and C1) and conserved (A2, B2, and C2) regions and the identity in percentage between two sequences.

The PfEMP1 family is encoded by *var* genes which include 56 *var* genes plus 3 small *var-like* genes (collectively called *var3*) [10, 27]. It has previously been shown that *var* genes can be subgrouped into three major groups (groups A, B, and C) and two intermediate groups B/A and B/C based on the conserved upstream sequence and genomic locations [16, 19]. The *var3* genes were also classified into group A based on the *N*-terminus sequences [19]. However, they are very different from the other *var* genes in sequence length and secondary structures of the encoded proteins. Earlier studies have found that the expression of group A PfEMP1s was associated with severe malaria with phenotypes of rosetting and adherence to endothelial receptors [15, 18, 32]. The DBL*α* (*α*-type Duffy binding-like) domains have been regarded as the molecular markers for classification of the sequence types associated with clinical diseases [13, 23, 31]. Further, previous studies have mainly been focused on the “typical” *var* genes, while the biological characteristics of the *var3* genes have not been extensively explored. Several studies suggested that *var3* was indeed expressed by clinical *P. falciparum* isolates as well as the laboratory-adapted parasite lines, which could be recognized by IgG from children from malaria-endemic regions [3, 30]. The three *var3* genes (PF3D7_0100300, PF3D7_0600400, and PF3D7_0937600) are located in subtelomeric regions of chromosome 1, 6, and 9, respectively. *Var3* genes are around 4 kb, and the secondary structures of the extracellular regions of the encoded proteins contain only one DBL*α* and one DBL*ε* domain, whereas the other PfEMP1 proteins are composed of multiple DBL domains and a cystine-rich interdomain region (CIDR). Thus, *var3* genes encoded “mini-PfEMP1s” contain a very compressed architecture and an atypical PfEMP1 structure (Fig. 1A). Further, the orthologs of the three *var3* genes are detected in nearly all parasite isolates [29], which are as conserved as the *var2csa* gene that encodes VAR2CSA associated with pregnancy-associated malaria. However, the function of the “mini-PfEMP1s” encoded by the *var3* genes is still not known. Further, the atypical intron activity of the *var3* genes suggested their expression might be under the control of a different mechanism from other *var* genes [7]. In this study, the transcription and expression of the individual *var3* genes were analyzed by quantitative RT-PCR, Western blot, and immunofluorescence.

## Methods

### Parasite culture

The *Plasmodium falciparum* 3D7 clone was cultured in human O^+^ RBC according to standard procedures [28]. The parasites were synchronized by three rounds of treatment at 4 h post-invasion with 5% sorbitol and parasites were harvested every 8 h, including 8, 16, 24, 32, 40, and 48 h post-infection.

### Transcription analysis of the *var3* genes with Quantitative RT-PCR

RNA at six time points after invasion was extracted using Trizol (Invitrogen, CA, USA) according to the manufacturer’s instructions. The extracted RNAs were treated with DNase I (TaKaRa, Dalian, China) to completely remove the genomic DNA. The RNA was reverse transcribed from Oligo(dT). The cDNAs were used as templates for quantitative RT-PCR with the specific primers; the information regarding the primer sequences is shown in Table 1. All primer sets had amplification efficiency >90%, which was determined automatically by the software. Quantitative RT-PCR was performed on an ABI PRISM^®^ 7500 Real-Time PCR System (Applied Biosystems) applying SYBR^®^ *Premix Ex Taq* ^TM^ (TaKaRa), and the internal control gene *seryl-tRNA* synthetase (PF3D7_1205100), which is stably expressed during the erythrocytic stage of the parasite, was used for normalization [21]. Further, two genes (PF3D7_0811600 and PF3D7_1361800) mainly expressed at 40–48 h p.i. were also included as external controls. Transcript levels relative to the average level of the internal control gene were calculated as 2^−ΔCt (var genes)^ [20]. The experiment was repeated three times and the transcription levels were represented by the mean values of the three experiments.

**Table 1.** The primer sequences of quantitative RT-PCR.

Gene name	Forward primer	Reverse primer
PF3D7_0937600	ATGGCACCGAAAA	GCCGCTTCCAATA
PF3D7_0100300	GGGATCATTATGGGAAGCAC	CGTTCTTGATTTCTACCATCGCA
PF3D7_0600400	ACGCCCAATTTCATGAT	TTCGGCATTTTCGTCAA

### Generation of recombinant protein and variant-specific antibodies

The gene fragments, named PF3D7_0100300 (amino acids 1-962), PF3D7_0600400 (amino acids 1-972), and PF3D7_0937600 (amino acids 1-1044) respectively, encoding the *N*-terminal regions (about 110 amino acids) of the three “mini-PfEMP1s” were selected as the target sequences. The three target sequences plus an ATS (acidic *C*-terminal segment) region were amplified from genomic DNA of the 3D7 *P. falciparum* clone. The ATS region was selected based on the complete conservation of the amino acid sequence (more than 90% identity between different PfEMP1 variants). The information regarding the primer sets is shown in Table 2. The amplified PCR products were cloned into the plasmid pET-28a (Qiagen, Düsseldorf, Germany) and pGEX-4T-1 (GE Healthsystems, Uppsala, Sweden) to construct recombinant plasmids. Expression and purification of His-tagged (ATS-His, PF3D7_0100300-His, PF3D7_0600400-His, and PF3D7_0937600-His) and GST-tagged (ATS-GST, PF3D7_0100300-GST, PF3D7_0600400-GST, and PF3D7_0937600-GST) recombinant proteins were carried out as described [20, 21, 26].

**Table 2.** The primer sequences for cloning the gene fragments encoding the recombinant proteins expressed in *E. coli.*

Gene name	Forward primer	Reverse primer
ATS	GTAGATATGATACGGATATT	TTTTGATGGCTCCAGAACAA
PF3D7_0937600	ATGGCACCGAAAAATGGA	TATTTTATCATTGTTACAA
PF3D7_0100300	ATGGCACTAAAAAAAG	CGTTCTTGATTTCTACCATCGCA
PF3D7_0600400	ATGGGATCAGATTATTCG	ACTATTACAATACGCTTC

Four female Wister rats received four immunizations with His-tagged recombinant proteins on days 0, 15, 30, 45, and 60 (100 μg/rat) emulsified in Freund’s complete (first immunization) and incomplete adjuvant (second to fourth immunizations). Simultaneously, three female New Zealand rabbits were immunized with ATS-His recombinant proteins. The experiments with laboratory animals were approved by the ethical committee of the Department of Agricultural Sciences, Jilin University. Specific IgG fractions in the immunized rats and rabbits were affinity-purified using the Protein G Sepharose^TM^ 4 Fast Flow (GE Healthcare) and the nProtein A Sepharose^TM^ 4 Fast Flow (GE Healthcare), respectively.

### Expression characterization of the native proteins

The infected erythrocytes at three developmental stages, including the ring, late trophozoite, and schizont stages, were purified by centrifugation on a gradient Percoll (GE Healthcare) as described (http://www.mr4.org/Portals/3/Methods_In_Malaria_Research-5theditionv5-2.pdf). Western blots and immunofluorescence assays were performed as described [11]. Briefly, the infected erythrocytes with parasites at three developmental stages, including the ring stage, late trophozoite stage, and schizont stage, were purified by gradient Percoll (GE Healthcare). For Western blotting, the total proteins of the infected erythrocytes were denatured in SDS-PAGE loading buffer (250 mM Tris, 1.92 M glycine, and 1% SDS) and subjected to electrophoresis under reducing conditions on an 8% polyacrylamide gel, and the proteins were transferred onto 0.2-μm nitrocellulose membranes (Bio-Rad, CA, USA). Membranes were blocked with 5% milk (Sigma, St. Louis, USA) for 1 h followed by incubation with the anti-ATS-His, anti-PF3D7_0100300-His, anti-PF3D7_0600400-His, and anti-PF3D7_0937600-His IgG (1:1000), respectively, as primary antibodies overnight at 4 °C. The membranes were further incubated with alkaline phosphatase conjugated mouse anti-rat IgG (Sigma, 1:20,000) for 1 h at 37 °C, BCIP/NBT substrate (Sigma) was added to the membranes and the reaction was terminated by rinsing in water after sufficient color development. To further confirm the expression of the three *var*3 genes, indirect immunofluorescence assays were performed. Thin smears of ring, late trophozoite, and schizont stage *P. falciparum*-infected erythrocytes were fixed with methanol at −80 °C for 5 min and blocked with PBS containing 5% nonfat milk at 37 °C for 1 h. The smears were then incubated with anti-ATS-His, anti-PF3D7_0937600-His, anti-PF3D7_0600400-His, and anti-PF3D7_0100300-His antibodies (1:50), followed by incubation with Alexa Fluor 488-conjugated goat anti-rat IgG (Invitrogen) (1:1000) at 37 °C for 1 h. Healthy rat IgG was used as a negative control antibody. The parasite nuclei were stained with DAPI (Roche, Basel, Switzerland) at room temperature for 5 min. High-resolution images were captured with a fluorescence microscope (Olympus, BX 53).

## Results and discussion

### Sequence analyses of the *var*3 genes

The encoded proteins by the three *var3* genes were divided into two regions based on sequence similarity. The most *N*-terminal regions of around 110 amino acids were variable, with 60%–67% identity between two sequences, while the internal regions were more similar with an identity of 88%–96% (Figs. 1A and 1B). The high similarity of this structurally distinct group of *var* genes indicated they shared a common origin and were generated by gene recombination [8, 22].

### Quantitative RT-PCR analysis of the *var3* genes in the 3D7 clone

**Figure 2.**
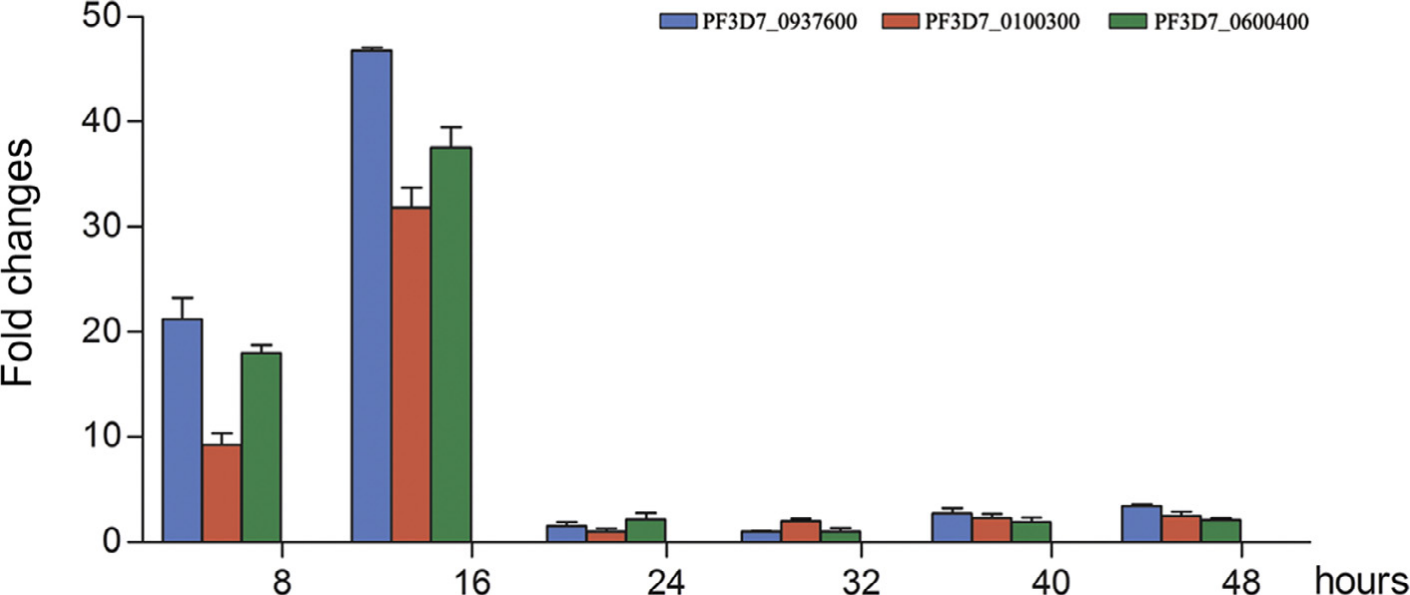
Transcriptions of the three *var3* genes in the *P. falciparum* 3D7 clone. The transcriptions of the three *var3* genes at 8, 16, 24, 32, 40, and 48 h p.i. are shown. Transcript levels relative to that of the internal control gene were calculated as 2^−ΔCt (var genes)^.

The transcriptions of the three *var3* genes in the *P. falciparum* 3D7 clone were determined by quantitative RT-PCR. The transcription of the three genes started early after erythrocyte invasion, which was in a similar way to other *var* genes [6], but with a relatively short time window. The transcription peaked at 16 h post-invasion and terminated immediately afterward (Fig. 2), which was obviously different from the other *var* genes [19], whose transcription could last until 30–36 h post-invasion [17, 30]. The two genes (PF3D7_0811600 and PF3D7_1361800) were found mainly expressed at 40 and 48 h p.i., respectively (data not shown). Further, earlier studies found that, in most cases, only one *var* gene was activated in an erythrocytic cycle [4, 24]. The data from this study found that the expression of the three *var3* genes might not follow the rule of mutual exclusion, since the encoded proteins were expressed in a major population of the parasites in the same culture, which will be further discussed later.

### The three *var-like* genes were differentially expressed in either the late trophozoite or schizont stages

**Figure 3.**
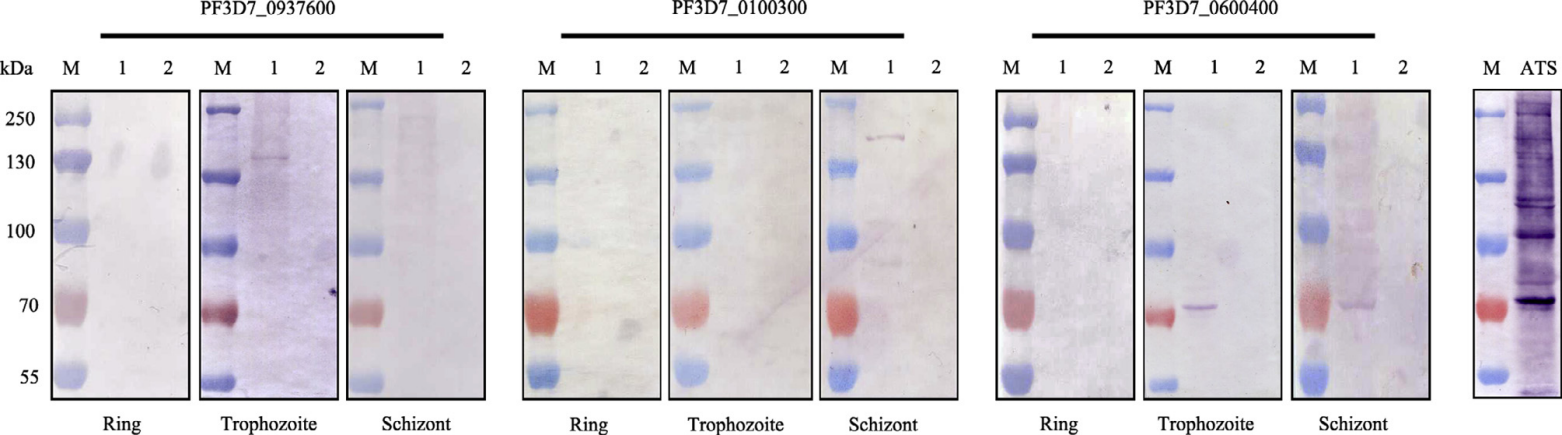
Western blot analysis of native proteins in three developmental stages. The expression of the native proteins encoded by the three *var*3 genes (PF3D7_0100300, PF3D7_0600400, and PF3D7_0937600) in the ring, trophozoite, and schizont stages were, respectively, detected by the variant-specific antibodies generated in rats. Lanes 1 and 2 represent results from infected red blood cells and uninfected red blood cells, respectively. A band of approximately 152 kDa was detected in the late trophozoite stage (24–30 h p.i.) with the antibody specific to PF3D7_0937600. A band of approximately 153 kDa was detected in the schizont stage (36–44 h p.i.) with the antibody specific to PF3D7_0100300. And a band of approximately 70 kDa was detected in both the trophozoite stage and schizont stage (24–44 h p.i.) for the PF3D7_0600400-specific antibody.

The expression of the *var3* genes were, in the first step, analyzed by Western blots using total proteins of infected erythrocytes as antigens, including the ring, late trophozoite, and schizont stage, respectively. The results showed that a single protein band was recognized by the variant-specific antibodies, except that with the anti-ATS antibody, which can literally recognize all PfEMP1 variants. Further, the *var*3 genes were found to be differentially expressed within three different time windows. The gene PF3D7_0600400 was expressed in both the late trophozoite stage and schizont stage, which was in a similar way to other *var* genes [14, 25]. However, the molecular size of the protein was much smaller than the theoretical value of the encoded protein. The reason is a mystery, since no sequence mutation was observed in either gDNA or cDNA. It was likely that the translation of the corresponding mRNA was terminated earlier controlled by an unknown mechanism, which needs to be further studied. PF3D7_0937600 was only expressed in the late trophozoite stage (24–32 h p.i.), but not in the schizont stage (36–44 h p.i.). In contrast, the protein encoded by PF3D7_0100300 could be detected in the schizont stage (36–44 h p.i.), but not in either the ring or late trophozoite stages (Fig. 3). Thus, the *var3* genes were activated in a similar way to other *var* genes, but the corresponding mRNAs were likely less stable. In an earlier study, Wang et al. found that proteins encoded by the *var3* genes were expressed on some of the unselected parasites from malaria patients [30]. Here, our data further showed that the three *var3* genes were indeed differentially expressed by the parasites, which strongly indicated that the “mini-PfEMP1” also participated in the immune evasion of *P. falciparum*.

**Figure 4.**
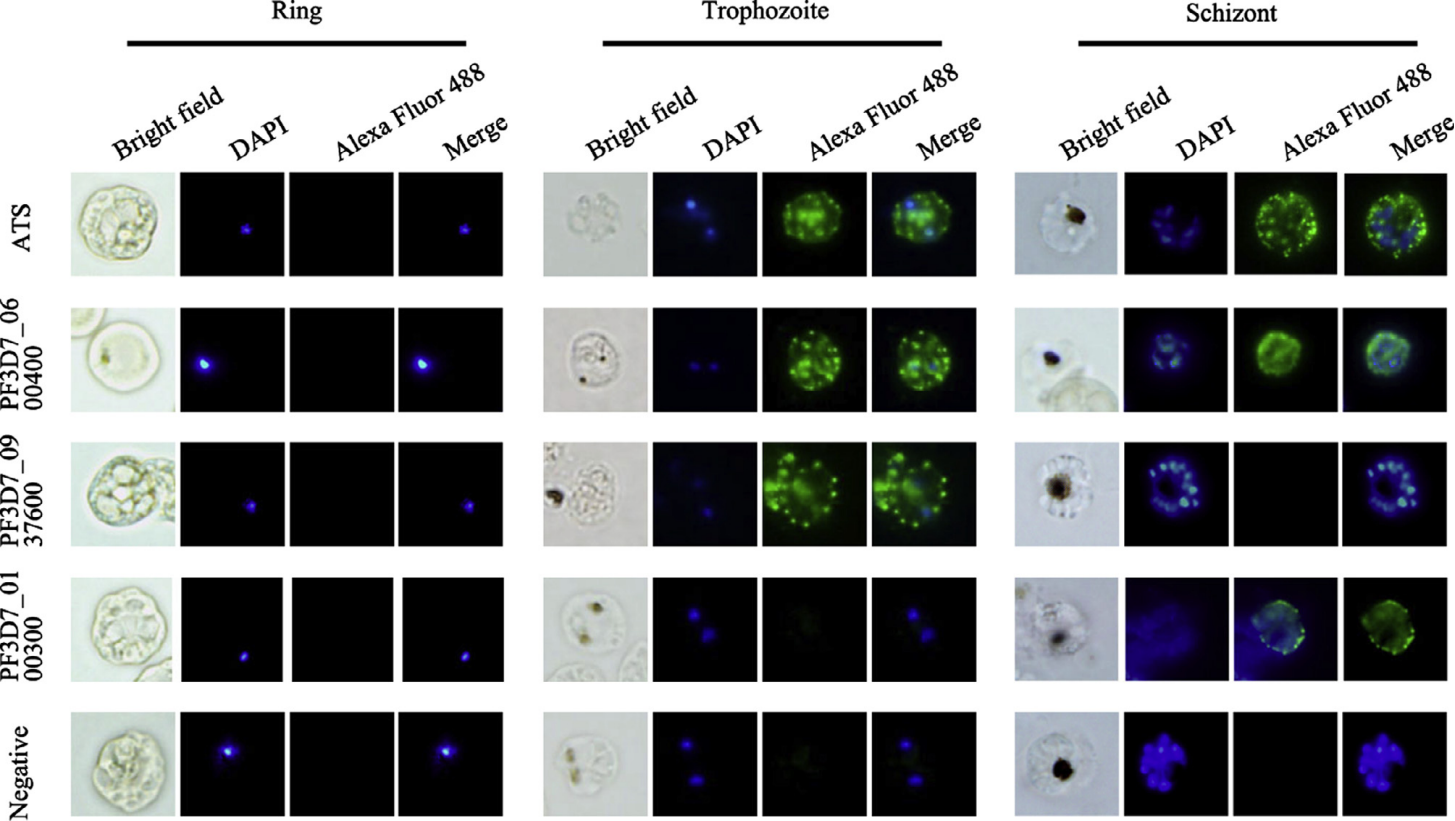
Immunofluorescence assays with variant-specific antibodies. An immunofluorescence assay (IFA) was performed using anti-PF3D7_0100300-His, anti-PF3D7_0600400-His, and anti-PF3D7_0937600-His IgG (1:50) as primary antibodies and anti-ATS IgG as the control. Specific staining of infected erythrocytes (IEs) is observed as punctuated fluorescence patterns over the IE surface using a secondary antibody labeled with Alexa 488 (green). The pre-immune sera of the immunized rats did not result in any surface reactivity with the IE. DAPI (5 μg/mL) staining of DNA in the nuclei is blue. The anti-ATS IgG and anti-PF3D7_0600400-His IgG stained IEs of both the trophozoite and schizont stages, the anti-PF3D7_0937600-His IgG only reacted with IEs of the trophozoite stage, while the anti-PF3D7_0100300-His IgG only reacted with IEs of the schizont stage.

To further confirm and localize the proteins expressed by the three *var3* genes in the infected erythrocytes, an immunofluorescence assay (IFA) was performed using the anti-PF3D7_0100300-His, anti-PF3D7_0600400-His, and anti-PF3D7_0937600-His IgGs as primary antibodies; meanwhile, an anti-ATS IgG was used as a control. The results clearly showed that the distribution of the proteins on the infected red blood cells was in a typical punctuated pattern of PfEMP1 [12]. The pre-immune IgG of the immunized rats did not show any surface reactivity with the pRBC. Further, as observed in the Western blot assays, PF3D7_0600400 was expressed in both the late trophozoite stage and schizont stage, while PF3D7_0937600 was only expressed in the late trophozoite stage and PF3D7_0100300 could only be detected in the schizont stage (Fig. 4). The punctuated patterns of the fluorescence of the proteins recognized with the variant-specific antibodies as well as the anti-ATS antibodies suggested that they followed the same translocation pathway as other PfEMP1s in the infected red blood cells. Thus, the three *var3* genes were transcribed at the same time but expressed in different time windows in the infected erythrocytes, indicating they are controlled by a distinct mechanism of posttranscriptional regulation, which requires further investigation.

In conclusion, the expression of the three atypical *var3* genes of the 3D7 *P. falciparum* clone was investigated by quantitative RT-PCR, Western blot, and immunofluorescence assay. It was found that the *var3* genes were differentially expressed during the erythrocytic stage of the parasite with features which differed from the typical *var* genes.

*Conflict of interest*: All authors have declared no conflict of interest.
